# Social withdrawal and anxiety-like behavior have an impact on zebrafish adult neurogenesis

**DOI:** 10.3389/fnbeh.2023.1244075

**Published:** 2023-10-16

**Authors:** Panagiotis Perdikaris, Paulina Prouska, Catherine R. Dermon

**Affiliations:** Laboratory of Human and Animal Physiology, Department of Biology, University of Patras, Patras, Greece

**Keywords:** social deficits, NMDAR hypofunction, MK-801, cell proliferation, neurogenesis, mGLuR5, zebrafish

## Abstract

**Introduction:**

Accumulating evidence highlights the key role of adult neurogenesis events in environmental challenges, cognitive functions and mood regulation. Abnormal hippocampal neurogenesis has been implicated in anxiety-like behaviors and social impairments, but the possible mechanisms remain elusive.

**Methods:**

The present study questioned the contribution of altered excitation/inhibition as well as excessive neuroinflammation in regulating the neurogenic processes within the Social Decision-Making (SDM) network, using an adult zebrafish model displaying NMDA receptor hypofunction after sub-chronic MK-801 administration. For this, the alterations in cell proliferation and newborn cell densities were evaluated using quantitative 5-Bromo-2′-Deoxyuridine (BrdU) methodology.

**Results:**

In short-term survival experiments. MK-801-treated zebrafish displayed decreased cell proliferation pattern within distinct neurogenic zones of telencephalic and preoptic SDM nodes, in parallel to the social withdrawal and anxiety-like comorbidity. BrdU+ cells co-expressed the pro-inflammatory marker IL-1β solely in MK-801-treated zebrafish, indicating a role of inflammation. Following the cessation of drug treatment, significant increases in the BrdU+ cell densities were accompanied by the normalization of the social and anxiety-like phenotype. Importantly, most labeled cells in neurogenic zones showed a radial glial phenotype while a population of newborn cells expressed the early neuronal marker TOAD or mGLuR5, the latter suggesting the possible involvement of metabotropic glutamate receptor 5 in neurogenic events.

**Discussion:**

Overall, our results indicate the role of radial glial cell proliferation in the overlapping pathologies of anxiety and social disorders, observed in many neuropsychiatric disorders and possibly represent potential novel targets for amelioration of these symptoms.

## 1. Introduction

A high rate of comorbidity between impairment in social functioning and anxiety characterizes many neuropsychiatric and neurological disorders (Allsop et al., 2014). Although, the etiology for the above comorbidity remains largely unknown, defects in adult hippocampal neurogenesis, including alterations in both progenitors’ proliferation as well as on the density of newly born neurons, have gained more attention as possible contributors to the co-existence of above pathologies (Malberg and Duman, 2003; Zhao et al., 2019; Gioia et al., 2022). More specifically, reduced number of hippocampal newly born neurons, attributed to decreased neuroblast proliferation, was observed in the R451C Neuroligin 3 knock-in mouse model of autism spectrum disorder (ASD) also characterized by reduced sociability (Gioia et al., 2022). Interestingly, prominent neurogenesis defects were observed in the prefrontal cortex and cerebellum of valproic acid-treated, non-human primates accompanied also by aberrant social interactions (Zhao et al., 2019). In addition, it has been shown that stressful experiences, including both acute and chronic stress, suppress proliferation and neurogenesis in the adult hippocampus of rodents (Malberg and Duman, 2003; Heine et al., 2004). Consistently, pharmacological rescue of adult neurogenesis in various rodent models, is known to contribute to the restoration of normal social behavior and amelioration of increased anxiety levels (Malberg and Duman, 2003; Gioia et al., 2022), providing further evidence for association between regulation of adult neurogenesis along with social deficits and anxiety-like behavior.

Adult neurogenesis is a multistage process, highly regulated by neuronal activity and experience (Zhao et al., 2008; Toda et al., 2019) and accumulating evidence indicates that neurotransmitters may influence the proliferation, differentiation, and survival of newborn cells (Platel et al., 2010; Song et al., 2012; Trinchero and Schinder, 2020). GABAergic and glutamatergic neurotransmission may regulate both the initial stages of neurogenesis by controlling the activation of quiescent neural stem progenitor cells (NSPCs) (Trinchero and Schinder, 2020) as well as later stages including differentiation, dendritic development, and synaptic integration of newborn neurons (Tozuka et al., 2005; Ge et al., 2006; Tashiro et al., 2006; Platel et al., 2010). N-methyl-D-aspartate receptors (NMDAR), besides their role in neuronal differentiation of adult-derived neural progenitor cells (NPCs) (Deisseroth et al., 2004) and/or *in vivo* survival of migrating neuroblasts (Platel et al., 2010), can also regulate cell proliferation since dentate gyrus NR1-knock out adult mice displayed decreased cell proliferation (Åmellem et al., 2021). In addition, the presence of excitation/inhibition imbalance at synaptic or circuit level, is considered a common pathophysiological mechanism implicated in the co-expression of social deficits and anxiety (Allsop et al., 2014) and also contributes to dysregulated neurogenesis in various psychiatric and neurological disorders (Kim et al., 2012; Zhao et al., 2019).

In the present study, we investigated the possible association between social deficits and comorbid anxiety with defects in proliferating NSPCs within diverse adult stem cell niches of the zebrafish brain (Grandel et al., 2006; Ampatzis et al., 2012; Makantasi and Dermon, 2014). More specifically, the mitotic activity was determined within 6 telencephalic and diencephalic neurogenic niches of adult zebrafish brain belonging to the Social-Decision Making (SDM) network, an evolutionary conserved network that regulates social behavior (O'Connell and Hofmann, 2011), using a zebrafish model displaying NMDAR hypofunction and characterized by deficits in social communication and increased anxiety levels. In addition, long-term survival experiments following the cessation of MK-801 treatment were performed to determine whether the effects on the behavioral phenotype and the accompanied alterations in mitotic activity and newborn cell densities were transient or long lasting.

## 2. Materials and methods

### 2.1. Animals

Wild-type, adult (3–4 months old) male zebrafish (*Danio rerio*) were selected from a stock of adult zebrafish, purchased by a local supplier, and housed in 40-L tanks equipped with biological filters (one fish/L), under constant aeration and a standard 12 h/10 h light/dark cycle, for at least 10 days prior to testing. Water quality including temperature (26 ± 1°C), pH value (7.0 ± 0.20) and total ammonia (≤0.01 mg/L) was continuously monitored, according to established standard of zebrafish care (Avdesh et al., 2012). All experimental procedures were approved by the Ethics Committee of University of Patras and were in accordance with the European Communities council directive (86/609/EEC) for the care and use of laboratory animals. All efforts were made to minimize animal suffering and to reduce the number of animals used.

### 2.2. MK-801 treatment and BrdU administration

(+)-MK-801 hydrogen maleate (MK-801) was purchased from Sigma Aldrich (St. Louis, Missouri, United States), dissolved in non-chlorinated water, and was administered to zebrafish via water immersion for 3 h per day, for 7 consecutive days at 1,349 ng/mL (4μΜ). Τhe dose of MK-801 was chosen based on a previous study in zebrafish (Perdikaris and Dermon, 2022). Control animals were exposed to the same conditions but treated with drug-free vehicle. Immediately after the daily treatment the animals returned to their home tanks and 2–3 h later, fish were allowed to have access to food.

**Figure 1:**
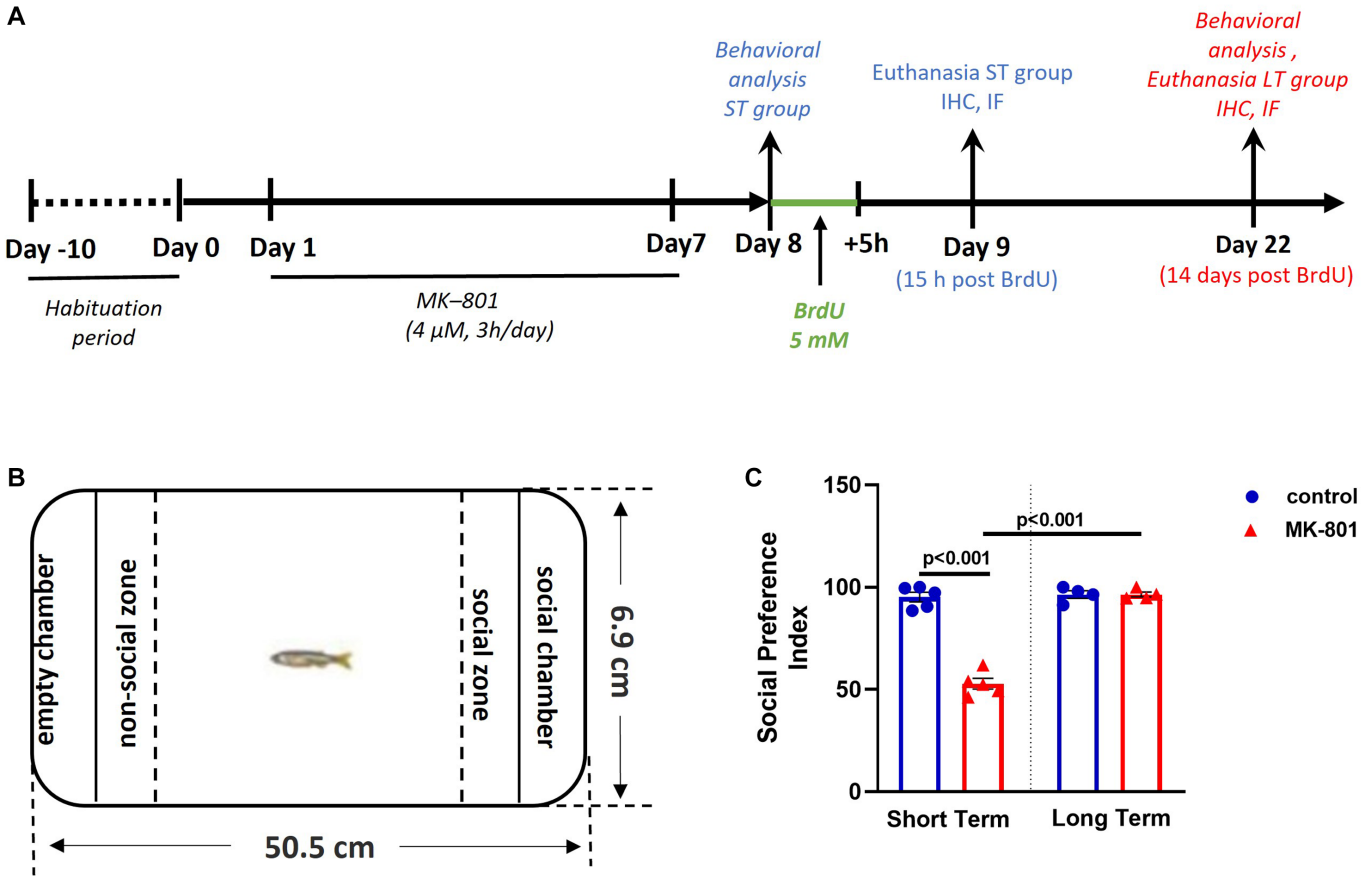
Social preference is impaired following MK-801 treatment, in short-term survival experiments but normalized in long-term experiments. **(A)** Schematic representation of the experimental protocol used. **(B)** Representation of the social preference test. **(C)** Social Preference Index of control and MK-801-treated zebrafish as estimated in the social preference test. Data are expressed as mean ± SEM. ST, short-term survival group; LT, long-term survival group. Control-ST group: *n* = 5, MK-801-ST group: *n* = 5, Control-LT group: *n* = 4, MK-801-LT group: *n* = 4.

At the 8th day of the experimental procedure, 24 h after MK-801 administration, zebrafish were individually subjected to a battery of behavioral tests as described below and then were immersed in fresh solution of 5-bromo-2′-deoxyuridine (ΒrdU) (Sigma-Aldrich, Deisenhofen, Germany) (5 mM, diluted in tank water) for 5 h, to label active cycling cells (Makantasi and Dermon, 2014). Fish were then allowed to survive for 15 h (short-term/ST survival group) and an additional group of fish were allowed to survive for 14 days (long-term/LT survival group), following BrdU administration at Day 8. The densities and phenotypes of newborn BrdU+ cells were assessed at Day 9 and Day 22 of the experimental protocol (Figure 1A).

### 2.3. Behavioral assays

Zebrafish were tested in a tank placed over or in the front of a lightbox, video-recorded either from above (Open Field Test, Social Preference Test) or laterally (Novel Tank Test), using a digital video camera and the analyses were performed using Ethovision XT9 (Noldus Information Technologies). Behavioral testing was conducted in a behavioral testing room under daylight conditions, at Day 8 and Day 22, between 10:00 and 15:00.

#### 2.3.1. Open-field test

The open-field test (OFT) was used to estimate zebrafish thigmotactic behavior and locomotor activity as described previously (Perdikaris and Dermon, 2022). Animals were introduced into the corner of a square arena (15 cm × 15 cm), divided into central and peripheral zone. Their swimming activity was analyzed for 6 min and the time spent in the central and peripheral zone was used to calculate the thigmotaxis index [% (T_peripheral zone_/T_arena_)] that served as an indicator of anxiety and fear-related responsiveness.

#### 2.3.2. Νovel tank test

The novel tank test (NTT) was performed in a standard 1.8 L trapezoid tank (17.7 top × 12.3 bottom × 15.5 height × 6.9 width) as previously described (Perdikaris and Dermon, 2022). Single fish were placed in this setup, divided in upper and lower zone, and video-recorded from lateral viewpoint for 6 min. The total time spent (sec) in the upper portion, the freezing duration and frequency and total distance swum (cm) were automatically quantified. Freezing was defined as a total cessation of movement for 1 s or longer, while at the bottom of the tank.

#### 2.3.3. Social preference test

The social preference (SP) of control and MK-801-treated adult fish was evaluated as described previously (Perdikaris and Dermon, 2022). Briefly, the experimental fish were placed in the central compartment of a three-compartment tank (50.5 cm length × 6.9 width × 15.5 height), separated by two transparent, perforated (1 mm holes) dividers, to acclimatize (150 s). A group of four unfamiliar fish, serving as “social stimulus,” was placed in one of the other two compartments (social chamber), whereas the second compartment remained empty (empty chamber). The time spent by the experimental fish in social and non-social zone was quantified and used to calculate the social preference index (SPI) [% (T_social zone_/T_social_ + T_non-social zone_)]. The social and non-social zone were defined within an area of 5 cm, immediately adjacent to the social and empty chamber. Total distance swum and mean velocity were also calculated.

### 2.4. Tissue preparation

After 15 h (*n* = 10) or 14 days (*n* = 8) post BrdU survival period, zebrafish were deeply anesthetized with 0,006% tricaine methane sulfonate (MS-222, Sigma-Aldrich, E10521) and intracardially perfused with saline followed by 4% paraformaldehyde (PFA, Sigma-Aldrich, Germany) in 0.1 M phosphate buffer (PB, pH = 7.4). Their brains were removed, post-fixed for 2 h in 4% PFA/PB and cryoprotected overnight in 20% sucrose in 4% PFA/PB at 4°C. Tissue was embedded in sectioning medium (Leica Biosystems, Buffalo Grove, United States) rapidly frozen in dry-ice-cooled isopentane (2-methylbutane; Sigma-Aldrich) at approximately −35°C and stored at −80°C. Coronal sections (20 μm thick) were cut in a Leica cryostat, collected in gelatin-coated slides, and stored at −80°C until used. After antigen retrieval with citrate buffer (10 mM Sodium citrate, 0.05% Tween 20, pH 6.0) at 80°C for 10 min, single or double immunohistochemistry was performed.

### 2.5. BrdU immunohistochemistry

For single-labeling experiments, sections were incubated in 1.5% H_2_O_2_ (Sigma-Aldrich) in 0.1 M PBS for 10 min in room temperature (RT) to inhibit endogenous peroxidase activity and then washed in phosphate-buffered saline (PBS, 0.01 M, pH 7.4). To visualize incorporated BrdU, DNA was denatured in 2 N HCl for 30 min at 37°C, followed by thorough washing in PBS and then sections were incubated in 1.5% H_2_O_2_ (Sigma-Aldrich) in PBS for 10 min in RT, to inhibit endogenous peroxidase activity. The sections were washed in PBS and non-specific sites were blocked with 5% (BSA; Sigma-Aldrich) and 1% normal horse serum (NHS) (Vector Laboratories) in Triton X-100/PBS (PBS-T, 0.5%) for 60 min at RT. The sections were incubated with mouse anti-BrdU (dilution 1:150, BD Biosciences Cat# 347580, RRID:AB_10015219) diluted in 1% BSA in PBS-T, for 16-18 h at 4°C. After thorough PBS rinses, the sections were incubated in biotinylated horse anti-mouse IgG secondary antibody (1:200, Vector Laboratories) in 1% BSA/PBS-T for 2.5 h at RT, washed in PBS-T, incubated with Vectastain Elite ABC reagent (1:100 A, 1:100 B; Vector Laboratories) in PBS-T for 1 h in the dark at RT and washed with PBS, followed by peroxidase-catalyzed polymerization of 3,3-diaminobenzidine (DAB; Vector Laboratories) to visualize the immunoreaction. The sections were then dehydrated with ethanol, cleared with xylene and cover slipped with Entellan (Merck, Darmstadt, Germany).

### 2.6. Phenotype of mitotically active and newborn cells

For double-labeling experiments, after the antigen retrieval step, sections were treated with 2 N HCl for 30 min at 37°C, and non-specific protein binding sites were blocked with 5% BSA and 1% NHS in PBS-T for 60 min at RT and then and incubated with a mixture of primary antibodies diluted in 1% BSA and 0.2% NHS in PBS-T, for 16-18 h at 4°C. The following primary antibodies were used: rabbit anti-mGluR5 (1:500, Millipore Cat# AB5675, RRID:AB_2295173), rabbit anti-Brain Lipid Binding Protein (BLBP) (1:800, Millipore Cat# ABN14, RRID:AB_10000325), rabbit anti-TUC4 (TOAD) (1:100, Millipore Cat# AB5454, RRID:AB_91876), rabbit anti-IL1β (1:250, Thermo Fisher Scientific Cat# P420B, RRID:AB_223478). After PBS washes, slides were incubated for 2.5 h at RT and in dark with a combination of secondary antibodies; AlexaFluor-488 donkey anti-mouse IgG (1:500, Thermo Fisher Scientific Cat# A-21202, RRID:AB_141607), AlexaFluor-555 donkey anti-mouse IgG (1:500, Thermo Fisher Scientific Cat# A-31570, RRID:AB_2536180), AlexaFluor-555 donkey anti-rabbit IgG (1:500, Thermo Fisher Scientific Cat# A-31572, RRID:AB_162543) and AlexaFluor-488 donkey anti-rabbit IgG (1:500, Thermo Fisher Scientific Cat# A-21206, RRID:AB_2535792) diluted in 1% BSA in PBS-T, washed thoroughly with PBS and cover-slipped with an aqueous fluorescent mounting medium (H-1700; Vector Laboratories).

To test the specificity of the primary antibodies used, additional negative controls were performed in adjacent sections, with the omission of the primary antibody or with application of secondary antisera mismatched for species. No labeling was observed in any case.

### 2.7. Microscopic observation and quantification of BrdU+ cells

The borders of the proliferation zones were determined using the zebrafish neuroanatomical atlas (Wullimann et al., 1996). The total number of BrdU+ cells was estimated in both short-term (*n* = 5/per experimental group) and long-term (*n* = 4 per experimental group) survival groups within 6 proliferation zones, using a Nikon Eclipse E800 optical microscope (Nikon, Tokyo, Japan) at 60X magnification. The proliferation zones and their total area analyzed were determined via a colored digital camera CFW-1308C (Scion Corp., United States), attached to a Nikon Eclipse E8 00 optical, with the aid of the ImageJ software. The density of BrdU+ cells in each proliferation zone was expressed as the fraction of BrdU+ cells per unit area (mm^2^). A minimum of 6 coronal sections per proliferation zone, per animal were used for quantification of BrdU+ cells in IHC staining. For the phenotypic analyses of the BrdU-positive cells, 2 to 3 sections per proliferation zone of interest per animal (*n* = 2 to 3 per experimental group per survival time) were quantified for each marker.

Images of single and double-labeling experiments were acquired using a colored digital camera CFW-1308C (Scion Corp., United States), attached to a Nikon Eclipse E800 optical and fluorescent microscope (Nikon, Tokyo, Japan). Each image consisted of a stack of optically sliced images, generated by NIH ImageJ software, and further used for identification of double-labeled cells. Images of BrdU-mGluR5 double labeling experiments were captured using a Leica SP8 confocal microscope.

### 2.8. Data analysis

Statistical analyses were performed using the statistical program IBM SPSS. For the analysis of behavioral and biochemical data, two-way analysis of variance (ANOVA) followed by independent samples *t*-test was used. The factors of variation were treatment and survival time. Data are presented as scatter plots using Graphpad Prism 6.0 (CA, United States) and expressed as mean ± standard error of the mean (SEM). Statistical significance was considered at *p* value < 0.05.

## 3. Results

### 3.1. MK-801 treatment induced transient impaired sociability and anxiety-like phenotype in zebrafish

The methodological approach displayed in Figure 1A was used to investigate the behavioral phenotype and BrdU+ cell densities in six adult zebrafish neurogenic zones, following MK-801 treatment, at two survival time points. The social preference of adult zebrafish was tested in the social preference (SP) test (Figure 1B). Two-way ANOVA indicated significant interaction between the effects of treatment and survival time on social preference index (SPI, Figure 1C) [*F*(1, 14) = 89.030, *p* ≤ 0.001], with a statistically significant simple main effect of treatment [*F*(1, 14) = 88.883, *p* ≤ 0.001] and survival period [*F*(1, 14) = 100.113, *p* ≤ 0.001].

More specifically, SPI was significantly decreased in “MK-801-ST” group compared to “control-ST” group [*t*(8) = 11.891, *p* ≤ 0.001] whereas no significant difference was observed between “control-LT” and “MK-801-LT” groups [*t*(6) = −0.008, *p* = 0.994]. As for the survival time condition, it was demonstrated that “MK-801-LT” group displayed increased SPI compared to “MK-801-ST” group [*t*(7) = −13.631, *p* ≤ 0.001], whereas no significant difference was observed between “control-ST” and “control-LT” groups [*t*(7) = −0.407, *p* = 0.696] (Figure 1C). Regarding locomotor activity, the two-way ANOVA indicated no significant interaction between the effects of treatment and survival period on mean velocity [*F*(1, 14) = 2.016, *p* = 0.178]. More specifically, there were no differences on mean velocity between “control-ST” and “MK-801-ST” or between “control-LT” and “MK-801-LT” groups [*t*(8) = 0.980, *p* = 0.372; *t*(6) = −1.253, *p* = 0.257 respectively] (data not shown). These results confirm that MK-801-treated fish exhibit a social withdrawal phenotype within the short-term survival period, not attributed to locomotor defects. Interestingly, this social phenotype was no longer present in the long-term survival group.

**Figure 2:**
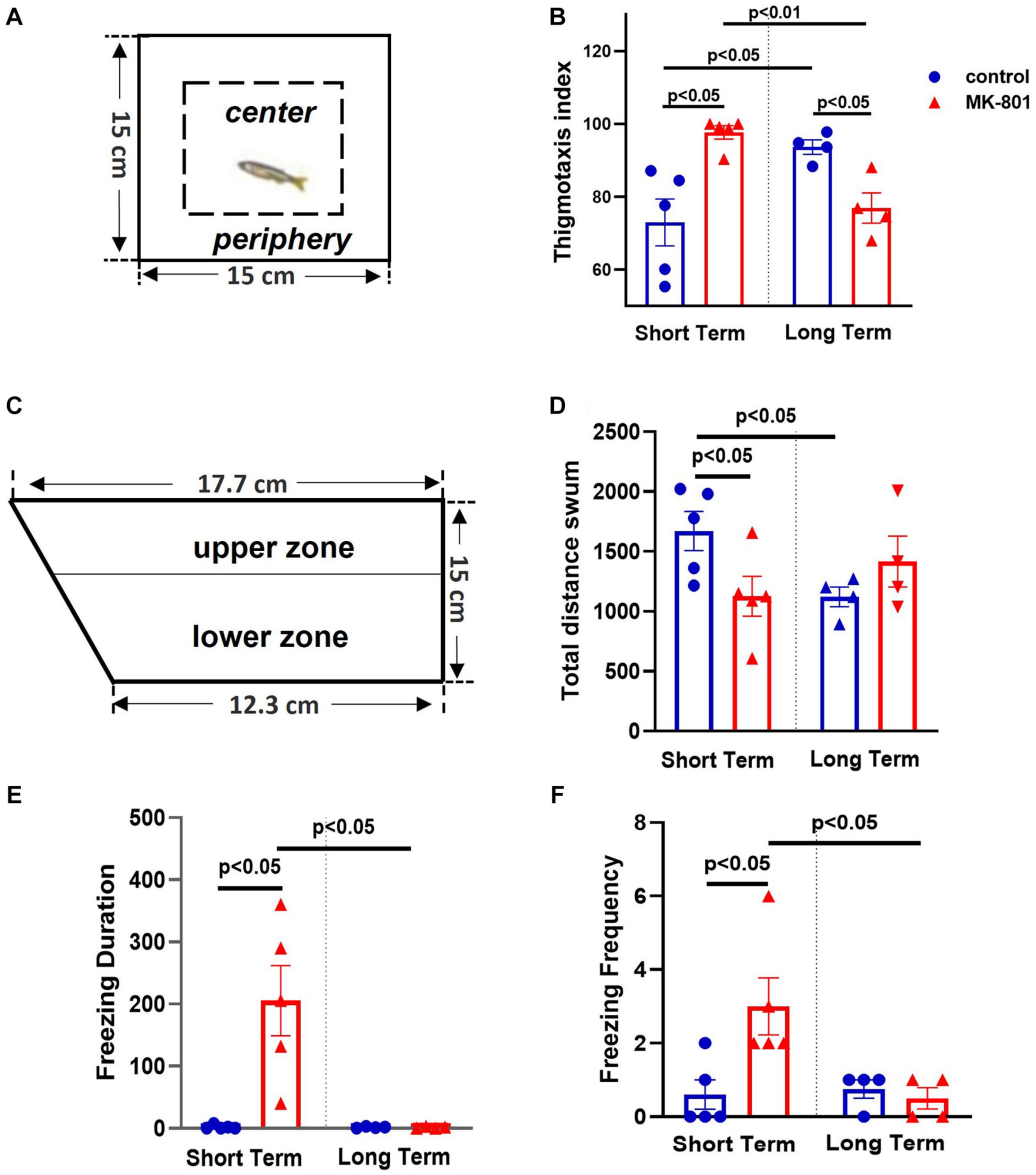
Elevated anxiety levels following MK-801 treatment, in short-term survival experiments but normalized 14 days post-BrdU administration. **(A)** Representation of the open field test (OFT), **(B)** Thigmotaxis index, **(C)** Representation of the novel tank test, **(D)** Total distance swum, **(E)** Freezing duration, **(F)** Freezing frequency. Data are expressed as mean ± SEM. Control-ST group: *n* = 5, MK-801-ST group: *n* = 5, Control-LT group: *n* = 4, MK-801-LT group: *n* = 4.

Anxiety-like state was determined using the open field test (OFT) and novel tank test (NTT). Regarding the OFT, (Figure 2A) the two-way ANOVA indicated significant interaction between the effects of treatment and survival period on thigmotaxis index [*F*(1, 14) = 23.145, *p* ≤ 0.001], whereas the simple main effects of treatment and survival time were not significant [treatment: *F*(1, 14) = 0.859, *p* = 0.370; survival period: *F*(1, 14) = 0.000, *p* = 0.995]. More specifically, while thigmotaxis index was significantly increased in “MK-801-ST” group compared to “control-ST” group [*t*(8) = −3.687, *p* = 0.016], it was significantly decreased in “MK-801-LT” compared to “control-LT” group [*t*(6) = 3.624, *p* = 0.011] (Figure 2B).

Regarding NTT, (Figure 2C), the two-way ANOVA indicated significant interaction between the effects of treatment and survival period on freezing duration [*F*(1, 14) = 10.079, *p* = 0.007], with a statistically significant simple main effect of treatment [*F*(1, 14) = 10.079, *p* = 0.007] and survival period [*F*(1, 14) = 10.474, *p* = 0.006]. That is, “MK-801-ST” group displayed increased freezing duration compared to “control-ST” group [*t*(8) = −3.600, *p* = 0.023] whereas no difference was observed between “control-LT” and “MK-801-LT” groups [*t*(6) = 0.253, *p* = 0.809] (Figure 2E). In line with these results, the two-way ANOVA indicated significant interaction between the effects of treatment and survival period on freezing frequency [*F*(1, 14) = 6.445, *p* = 0.024]. “MK-801-ST” group displayed higher frequency of freezing bouts compared to “Control-ST” group [*t*(8) = −2.753; *p* = 0.025], but no difference was observed between “control-LT” and “MK-801-LT” groups [*t*(6) = 0.655; *p* = 0.537] (Figure 2F). Regarding the total time spent in the upper zone, the two-way ANOVA indicated a significant effect of treatment [*F*(1, 14) = 6.508, *p* = 0.023] but not of survival period [*F*(1, 14) = 0.008, *p* = 0.929] nor interaction [treatment × survival period, *F*(1, 14) = 0.024, *p* = 0.880] (data not shown). Total distance swum was also quantified as a readout of exploratory activity. Two-way ANOVA indicated a significant interaction between the effects of treatment and survival period on total distance swum [*F*(1, 14) = 6.471, *p* = 0.023], but no significant effect of treatment [*F*(1, 14) = 1.297, *p* = 0.274] or survival period [*F* (1, 14) = 0.007, *p* = 0.934] was observed. Independent samples *t*-test revealed that “MK-801-ST” group swum decreased distance compared to “control-ST” group [*t*(8) = 2.376, *p* = 0.045], a further possible indication of reduced exploration in a novel environment, whereas no difference was observed between “control-LT” and “MK-801-LT” groups [*t*(6) = −1.293; *p* = 0.244] (Figure 2D).

Overall, our results indicate that social dysfunction and increased anxiety levels in MK-801-treated zebrafish were transiently expressed at day 8 but were not present at day 22.

### 3.2. Differential regulation of BrdU+ cell densities following MK-801 administration

The density (number/mm^2^) of BrdU+ cells within 6 proliferation zones of the SDM network was estimated, following MK-801 treatment, at 15 h (short-term survival group) and 14 days (long-term survival group) post-BrdU. As expected, in most cases, a significant increase in the number of BrdU+ cells characterized both long survival groups compared to their matched short-term controls, possibly due to further divisions of cycling BrdU+ cells. Indeed, is known that in addition to postmitotic cells, BrdU labeling is diluted to half at each cell cycle and allows the detection of approximately three cell cycles maximum (Hayes and Nowakowski, 2002).

#### 3.2.1. MK-801 effect on the neurogenic zones of dorsal telencephalic areas

##### 3.2.1.1. Medial zone of the dorsal telencephalic area (Dm)

**Figure 3:**
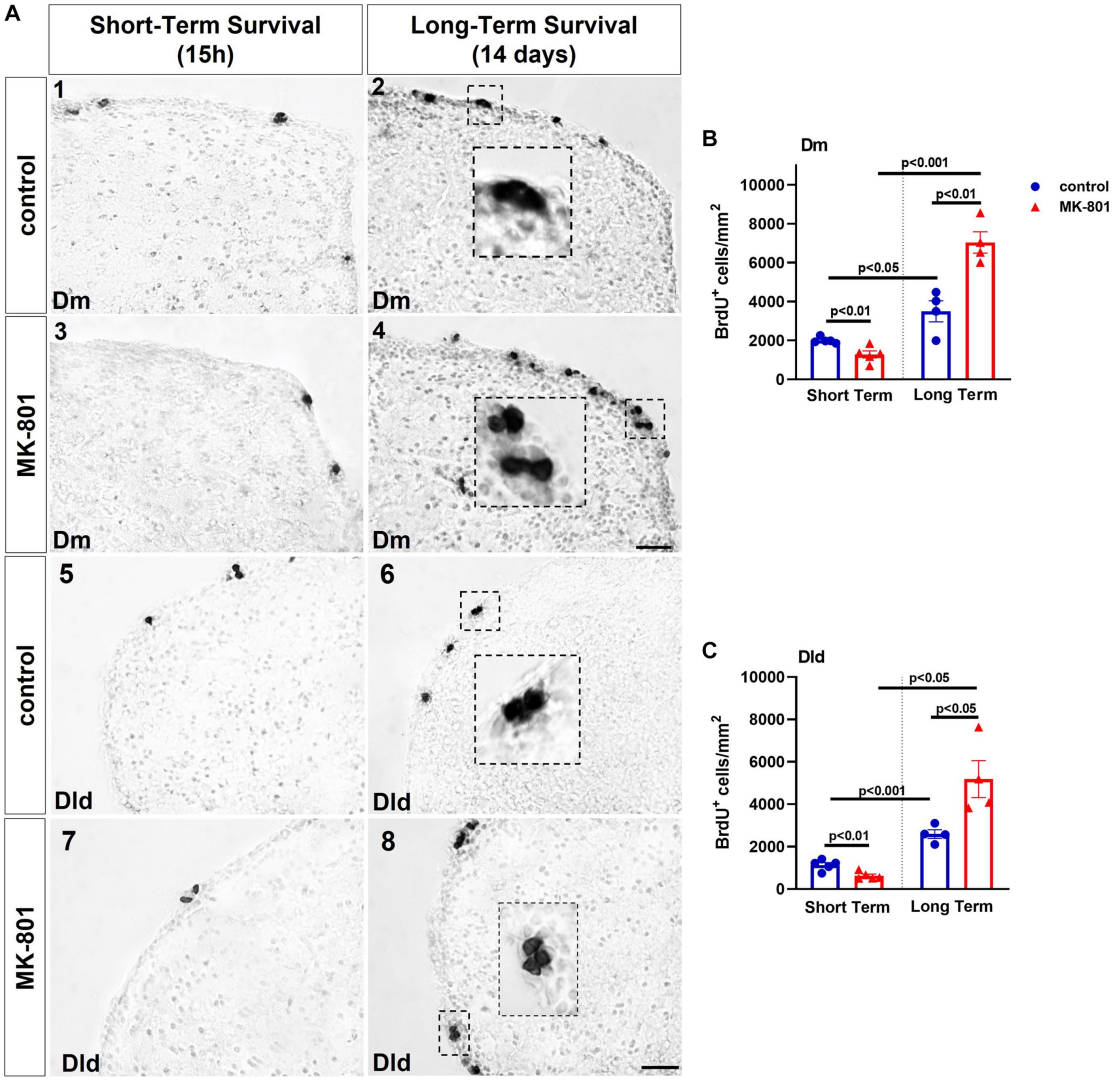
MK-801-treated zebrafish are characterized by decreased cell proliferation within the neurogenic niches of the dorsal telencephalon. **(A)** Representative immunohistochemical staining images of medial zone of the dorsal telencephalic area (Dm) (1–4) and dorsal lateral zone of the dorsal telencephalic area (Dld) (5–8) showing the distribution of BrdU-positive cells 15 h (short-term survival; 1, 3, 5, 7) and 14 days (long-term survival; 2, 4, 6, 8) post-BrdU administration. **(B,C)** Density of BrdU+ cells in **(B)** Dm and **(C)** Dld of control and MK-801-treated zebrafish counted at 15 h or 14 days post-BrdU administration. Notice the BrdU+ cells appearing in clusters in long-term survival groups. Data are expressed as mean ± SEM. Scale bar: 25 μm. Control-ST group: *n* = 5, MK-801-ST group: *n* = 5, Control-LT group: *n* = 4, MK-801-LT group: *n* = 4. ST, short-term survival group; LT, long-term survival group.

Two-way ANOVA indicated a significant interaction between the effects of treatment and survival period on total number of BrdU+ cells in the proliferation zone of Dm [*F*(1, 14) = 35.531, *p* ≤ 0.001], with a statistically significant simple main effect of treatment [*F*(1, 14) = 15.635, *p* = 0.001] and survival period [*F*(1, 14) = 103.479, *p* ≤ 0.001]. More specifically, “MK-801-ST” group displayed a statistically significant decrease on the number of BrdU+ cells (mitotically active cells) compared to “control-ST” group [*t*(8) = 3.668, *p* = 0.006]. Following 2 weeks of survival time, both LT groups showed significantly increased densities of BrdU+ cells (newborn cells) in “control-LT” group compared to “control-ST” group [*t*(7) = −3.119, *p* = 0.017], as well as in “MK-801-LT” group compared to “MK-801-ST” group [*t*(7) = −10.935, *p* ≤ 0.001]. Importantly, “MK-801-LT” group included significantly higher number of labeled cells compared to “control-LT” group [*t*(6) = −4.580, *p* = 0.004] (Figures 3A,B).

##### 3.2.1.2. Dorsal lateral zone of the dorsal telencephalic area (Dld)

Similarly, two-way ANOVA indicated a significant interaction between the effects of treatment and survival period on total number of BrdU+ cells in the proliferation zone of Dm [*F*(1, 14) = 14.964, *p* = 0.002], with a statistically significant simple main effect of treatment [*F*(1, 14) = 6.813, *p* = 0.021] and survival period [*F*(1, 14) = 56.652, *p* ≤ 0.001]. More specifically, “MK-801-ST” group displayed a statistically significant decrease on the density of BrdU+ cells compared to “control-ST” group [*t*(8) = 3.716, *p* = 0.006], whereas the increment in the density of BrdU+ cells was significant in “MK-801-LT” group compared to “control-LT” group [*t*(6) = −2.899, *p* = 0.027]. The density of BrdU+ cells of both LT groups was increased compared to equivalent ST groups [“control-ST” group compared to “control-LT” group: (*t*(7) = −6.654, *p* ≤ 0.001), “MK-801-ST” group compared to “MK-801-LT” group: (*t*(7) = −5.218, *p* = 0.013) (Figures 3A, C)].

#### 3.2.2. MK-801 effect on the neurogenic zones of ventral telencephalic areas

##### 3.2.2.1. Ventral nucleus of the ventral telencephalic area (Vv)

**Figure 4:**
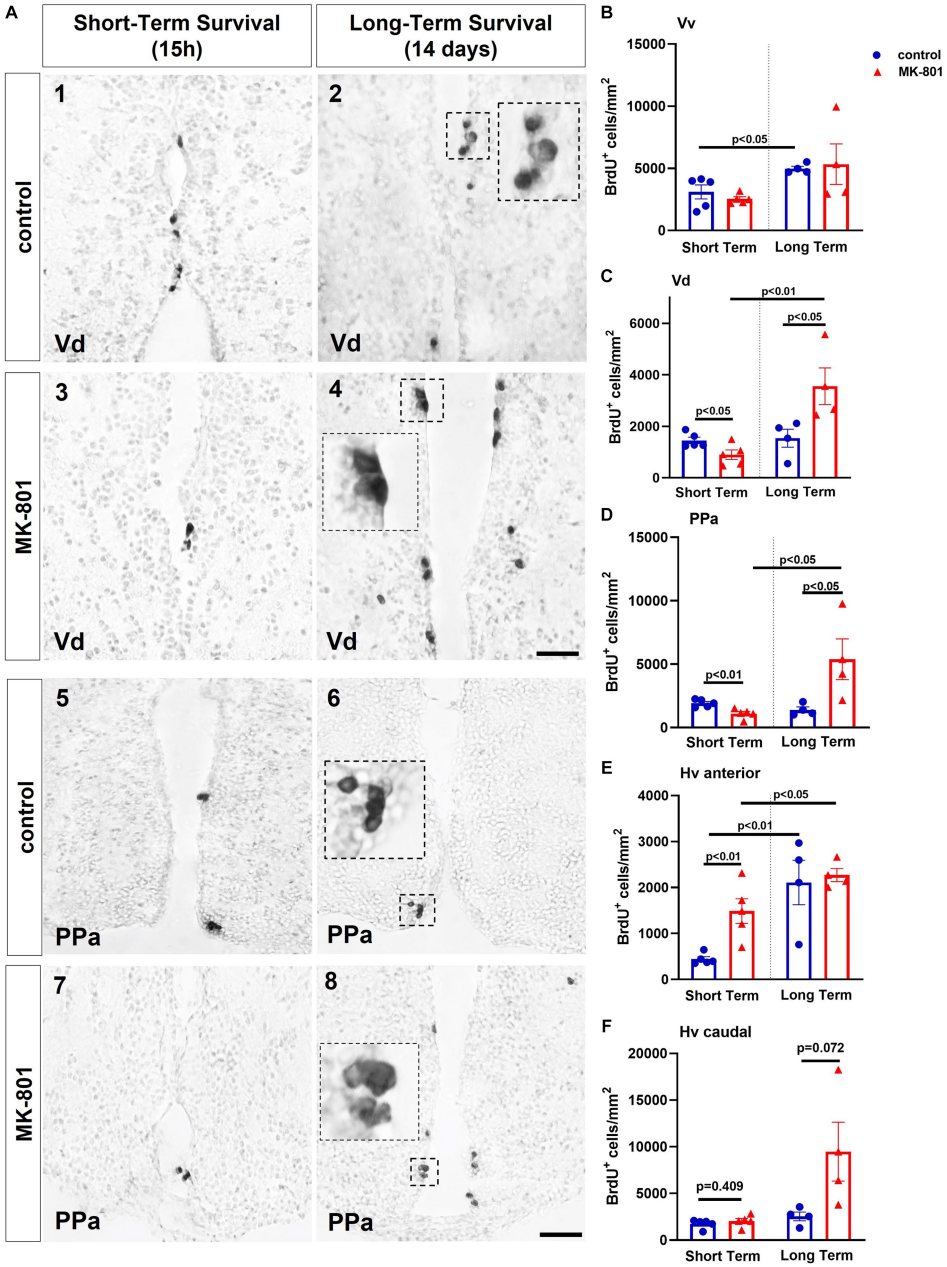
MK-801-treated zebrafish are characterized by decreased cell proliferation within the neurogenic niches of ventral telencephalon and ventral zone of the periventricular hypothalamus (Hv). **(A)** Representative immunohistochemical staining images of dorsal zone of the ventral telencephalic area (Vd) (1–4) and parvocellular preoptic nucleus, anterior part (PPa) (5–8) showing the distribution of BrdU-positive cells 15 h (short-term survival; 1, 3, 5, 7) and 14 days (long-term survival; 2, 4, 6, 8) post-BrdU administration. **(B–E)** Density of BrdU+ cells in **(B)** ventral zone of the ventral telencephalic area (Vv), **(C)** Vd, **(D)** PPA, **(E,F)** Hv anterior and posterior part of control and MK-801-treated zebrafish, counted at 15 h or 14 days post-BrdU administration. Notice BrdU+ cells appearing in clusters in long-term survival groups. Data are expressed as mean ± SEM. Scale bar: 25 μm. Control-ST group: *n* = 5, MK-801-ST group: *n* = 5, Control-LT group: *n* = 4, MK-801-LT group: *n* = 4. ST, short-term survival group; LT, long-term survival group.

Two-way ANOVA indicated no significant interaction between the effects of treatment and survival period on the total number of BrdU+ cells in Vv [*F*(1, 14) = 0.335, *p* = 0.572] nor on simple main effect of treatment [*F*(1, 14) = 0.017, *p* = 0.897], whereas only a significant effect of survival period [*F*(1, 14) = 8.590, *p* = 0.011] was observed. Simple main effects analysis showed that “control-LT” group displayed increased total number of BrdU+ cells compared to “control-ST” group [*t*(7) = −2.833, *p* = 0.026] (Figure 4B).

##### 3.2.2.2. Dorsal nucleus of the ventral telencephalic area (Vd)

Two-way ANOVA indicated a significant interaction between the effects of treatment and survival period on the total number of BrdU+ cells in the proliferation zone of Vd [*F*(1, 14) = 12.065, *p* = 0.004]. Moreover, only the simple main effect of survival period was statistically significant [*F*(1, 14) = 13.726, *p* = 0.002]. “MK-801-ST” group displayed a statistically significant decrease on the density of BrdU+ cells compared to “control-ST” group [*t*(8) = 2.465, *p* = 0.0039], whereas within the long-term survival period the density of BrdU+ cells was significantly increased after MK-801 treatment compared to control [*t*(6) = −2.549, *p* = 0.044]. A significant increase in the “MK-801-LT” BrdU+ cell density compared to “MK-801-ST” group was evident [*t*(7) = −4.036, *p* = 0.005] (Figures 4A,C).

#### 3.2.3. MK-801 effect on neurogenic zones of preoptic and hypothalamic areas

##### 3.2.3.1. Parvocellular preoptic nucleus, anterior part (PPa)

Two-way ANOVA indicated a significant interaction between the effects of treatment and survival period on total number of BrdU+ cells in the proliferation zone of PPa [*F*(1, 14) = 11.115, *p* = 0.005], with a statistically significant simple main effect of treatment [*F*(1, 14) = 4.819, *p* = 0.045] and survival period [*F*(1, 14) = 6.804, *p* = 0.021]. More specifically, “MK-801-ST” group displayed a statistically significant decrease on the density of BrdU+ cells compared to “control-ST” group [*t*(8) = 3.806, *p* = 0.005], whereas a significant increment on the density of BrdU+ cells was observed in the “MK-801-LT” group compared to “control-LT” group [*t*(6) = −2.464, *p* = 0.049]. A significant rebound was also observed in the density of BrdU+ cells in the “MK-801-LT” group compared to “MK-801-ST” group [*t*(7) = −3.016, *p* = 0.019] (Figures 4A,D).

##### 3.2.3.2. Ventral zone of the periventricular hypothalamus (Hv)

The Hv nucleus was divided in anterior (cross section 131–136) and posterior part (cross section 149–158) according to Wullimann et al. (1996). Two-way ANOVA indicated no significant interaction between the effects of treatment and survival period on the density of BrdU+ cells in the proliferation zone of anterior Hv [*F*(1, 14) = 2.684, *p* = 0.124], but a statistically significant simple main effect of treatment [*F*(1, 14) = 4.996, *p* = 0.042] and survival period [*F*(1, 14) = 20.608, *p* ≤ 0.001]. In contrast to the other brain regions studied, the density of BrdU+ cell density was significantly increased in “MK-801-ST” group compared to “control-ST” group [*t*(8) = −3.828, *p* = 0.005] whereas no significance difference was observed between “control-LT” and “MK-801-LT” group [*t*(6) = −0.320, *p* = 0.760]. However, BrdU+ cell densities were significantly increased in “control LT” compared to “control-ST” group [*t*(7) = −3.893, *p* = 0.006], as well as in “MK-801-LT” group compared to “MK-801-ST” group [*t*(7) = −2.384, *p* = 0.049] (Figure 4E). Regarding the proliferation zone of the posterior Hv, two-way ANOVA indicated a significant interaction between the effects drug treatment X survival period on the number of BrdU+ cells [*F*(1, 14) = 5.518, *p* = 0.034], with a statistically significant simple main effect of treatment [*F*(1, 14) = 6.591, *p* = 0.022] and survival time [*F*(1, 14) = 8.507, *p* ≤ 0.011]. Further analysis with independent samples *t*-test demonstrated no significant difference between “control-ST” and “MK-801-ST” groups [*t*(8) = −0.872, *p* = 0.409] or between “control-LT” and “MK-801-LT” groups [*t*(6) = −2.181, *p* = 0.072] (Figure 4F).

### 3.3. Phenotype of proliferating/newborn cells within adult neurogenic zones

#### 3.3.1. Telencephalic and diencephalic BrdU+ cells express radial glia marker, BLBP

**Figure 5:**
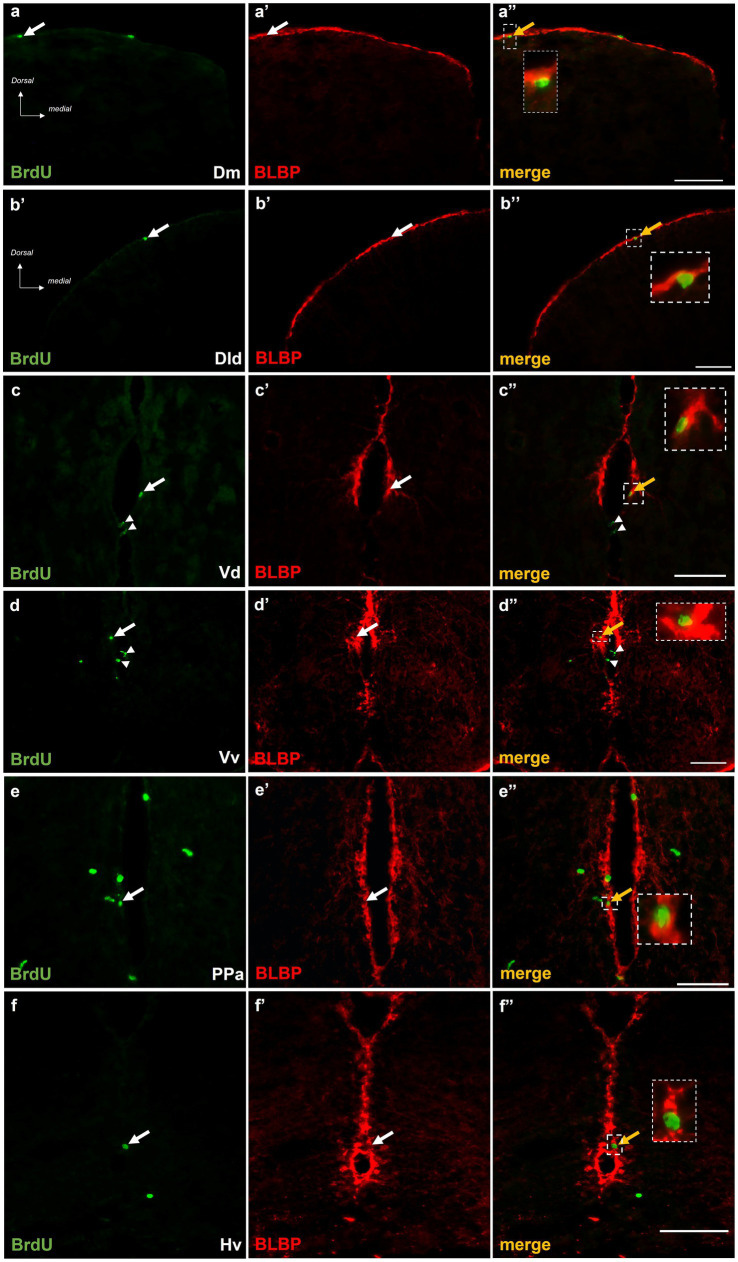
BrdU+ cells in telencephalic and diencephalic proliferation zones of SDM network in short-term survival group, display radial glia phenotype. Immunofluorescent microphotographs of selected transverse sections showing BrdU+ proliferative cells (green) in relation to BLBP-expressing cells (red) in the neurogenic zone of **(a–a”)** Dm, **(b–b”)** Dld, **(c–c”)** Vd, **(d–d”)** PPa, and **(e–e”)** Hv anterior part, 15 h after BrdU administration. Yellow arrows and boxed areas indicate examples of colocalization, arrowheads point to single positive cells. Microphotographic images are representative of control-short term survival group. Scale bar: 50 μm.

The phenotype of BrdU+ cells was questioned in the short and long survival time points in double-labeling experiments, using the radial glia marker BLBP (März et al., 2010). At survival time 15 h post-BrdU, a significant percentage of mitotically active cells (ranging from 45 to 60% of the BrdU+ cells) within the proliferation zones of Dm (Figures 5a–a”), Dld (Figures 5b–b”), and Vd (Figures 5c–c”) displayed radial glia phenotype, in “control-ST” and “MK-801-ST” survival groups. In contrast, a smaller percentage of approximately 15 to 20% of the BrdU+ cells in the neurogenic zones of PPa (Figures 5e–e”) and Hv (Figures 5f–f”) were found to express BLBP in “control-ST” and “MK-801-ST” group. Our results also demonstrated the existence of BrdU+/BLBP-negative proliferating cells (Figures 5d–d”) which most likely represent committed progenitors (proliferating neuroblasts) (Diotel et al., 2020).

#### 3.3.2. Metabotropic glutamate receptor 5 is expressed in BrdU+ cells

**Figure 6:**
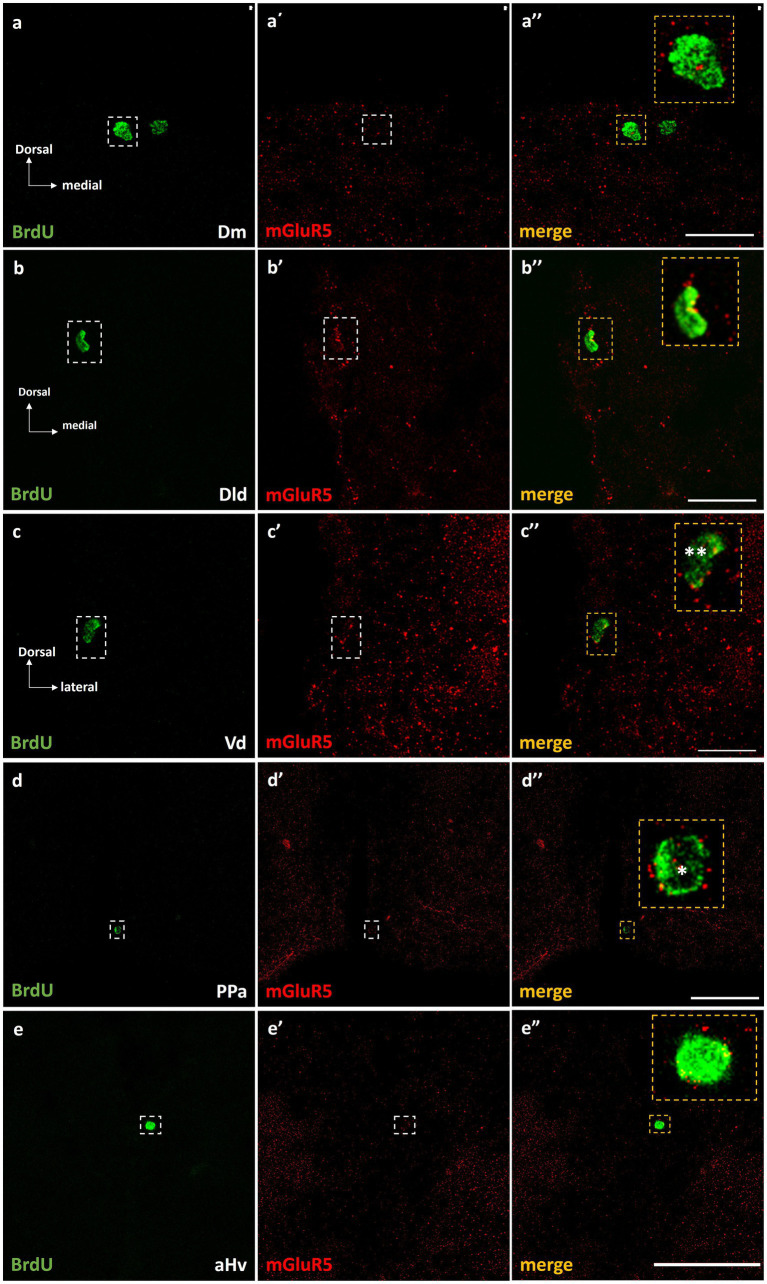
mGluR5 is expressed on BrdU+ cells in telencephalic and diencephalic proliferation zones of SDM network, in the short-term survival group. Immunofluorescent microphotographs of selected transverse sections showing the localization of mGluR5 (red) on BrdU+ proliferative cells (green) in the neurogenic zone of **(a–a”)** Dm, **(b–b”)** Dld, **(c–c”)** Vd, **(d–d”)** PPa, and **(e–e”)** Hv anterior part, 15 h after BrdU administration. Yellow boxed areas indicate examples of colocalization. Notice both the cell surface (*) and/or nuclear localization (**) of mGluR5. Microphotographic images are representative of control-short term survival group. Scale bar: 50 μm.

Importantly, double immunofluorescent experiments 15 h post-BrdU, demonstrated the localization of mGluR5 immunopositive puncta on approximately 10–15% of the BrdU+ cells, in the proliferation zones of Dm (Figures 6a–a”), Dld (Figures 6b–b”), Vd (Figures 6c–c”), PPa (Figures 6d–d”), and Hv (Figures 6e–e”), in both groups.

#### 3.3.3. Interleukin-1 beta is expressed in neurogenic niches of MK-801-treated zebrafish

**Figure 7:**
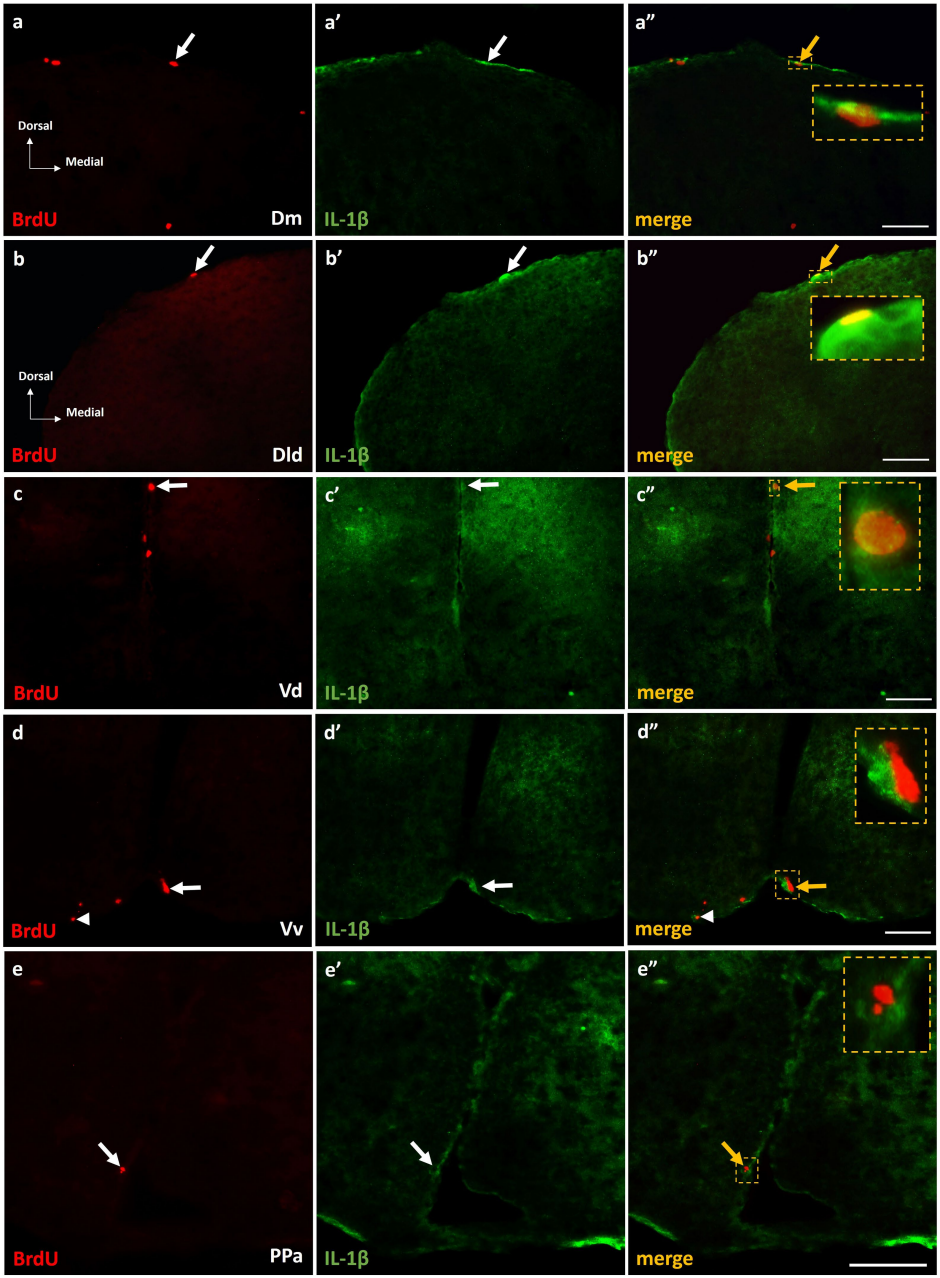
IL-1β expression on BrdU+ cells in telencephalic proliferation zones of SDM network, in the short-term survival group. Immunofluorescent microphotographs of selected transverse sections showing the expression of IL-1β (green) on BrdU+ proliferative cells (red) in the neurogenic zone of **(a–a”)** Dm, **(b–b”)** Dld, **(c–c”)** Vd, **(d–d”)** Vv, and **(e–e”)** PPa, 15 h after BrdU administration. Yellow arrows and boxed areas indicate examples of colocalization. Microphotographic images are representative of MK-801-short term survival group. Scale bar: 50 μm.

The expression of IL-1β within the proliferation zones of adult zebrafish brain, following MK-801 treatment, was questioned since previous studies demonstrated that excessive IL-1β expression within areas of the SDMN, related with the asocial and anxiety-like phenotype of MK-801-treated fish (Perdikaris and Dermon, 2023). Interestingly, at short survival experiments, approximately 5–10% of the BrdU+ cells were found to express IL-1β within the telencephalic and diencephalic neurogenic zones of Dm (Figures 7a–a”), Dld (Figures 7b–b”), Vd (Figures 7c–c”), Vv (Figures 7d–d”), and PPa (Figures 7e–e”) in the “MK-801-ST.” In contrast, in “control-ST” group (data not shown) IL-1β was not found to be expressed on BrdU+ cells. In addition, in the “MK-801-ST” group, IL-1β expressing cells were also found in close proximity to BrdU+ proliferating cells (Figures 7d–d”, arrowheads), indicating a possible influence of IL-1β in the mitotic activity within adult neurogenic zones.

#### 3.3.4. Phenotype of newborn cells, 14 days post-BrdU

**Figure 8:**
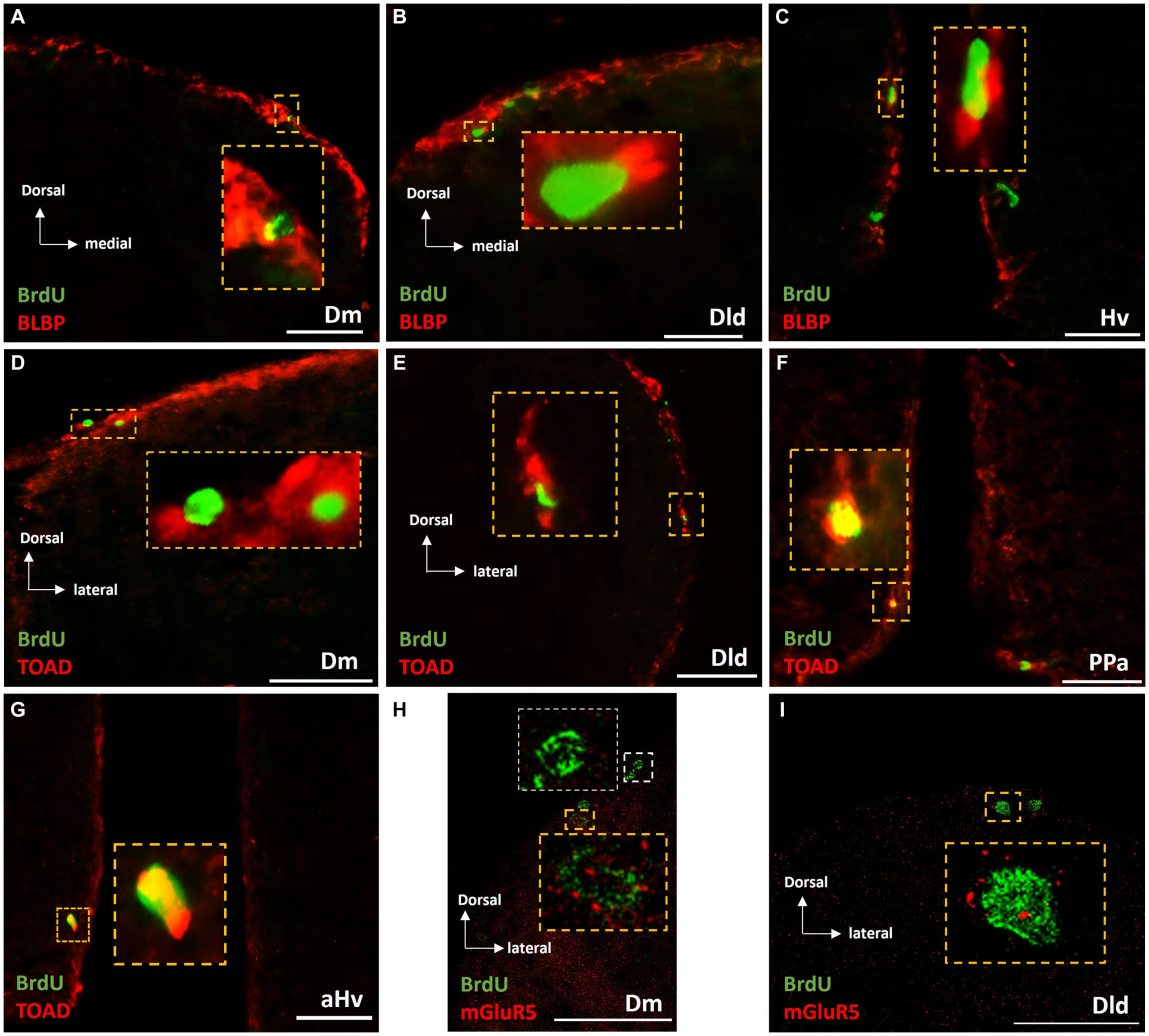
Phenotypic characterization of BrdU+ cells in telencephalic and diencephalic proliferation zones of SDM network, in the long-term survival group. Immunofluorescent microphotographs of selected transverse sections showing the expression of BLBP (red), TOAD (red) and mGluR5 (red) on BrdU+ proliferative cells (green) in the neurogenic zone of **(A,D,H)** Dm, **(B,E,I)** Dld, **(F)** PPa, and **(C,G)** Hv anterior part, 15 days after BrdU administration. Yellow boxed areas indicate examples of colocalization. White box indicates a BrdU+ cell not expressing mGluR5. Microphotographic images are representative of control **(A–G)** and MK-801 **(H,I)** long-term survival group. Yellow arrows indicate examples of colocalization. Scale bar: 50 μm.

Long survival experiments of “control” and “MK-801-treated groups, showed a similar percentage of BrdU+ /BLBP + double labeled cells, ranging from 40 to 50% of BrdU+ cells within the neurogenic zones of Dm (Figure 8A), Dld (Figure 8B) and Vd (data not shown), suggesting their commitment to a radial glia phenotype. In the neurogenic zones of PPa (data not shown) and Hv (Figure 8C), a lower percentage of BrdU+ cells were found to express BLBP (approximately 5–10%).

In addition, the early neuronal marker TOAD was found to be expressed by a significant population of newborn cells in the long-term survival of both “control” and “MK-801-treated” groups, indicating their neuronal commitment. Specifically, in both “control-LT” and “MK-801-LT” groups, 30–35% of BrdU+ cells in Dm (Figure 8D), Dld (Figure 8E) and Vd (data not shown) as well as 40–45% in the neurogenic zones of PPa (Figure 8F) and Hv (Figure 8G) were found to co-express TOAD.

While a percentage of BrdU+ cells was found to express IL-1β, in MK-801-ST experimental animals, this was not the case for MK-801-LT group, since no significant levels of IL-1β expression were detected in the aforementioned proliferation zones and low IL-1β expression characterized brain parenchyma.

Importantly, 14 days post-BrdU, both “MK-801-LT” and “control-LT” groups, showed BrdU+/mGluR5+ expressing cells within dorsal and ventral telencephalic proliferation zones, representing 15–20% of the BrdU+ cells (Figures 8H,I; Supplementary Figure S1). In rare instances, mGluR5+/BrdU+ cells were also found in the neurogenic niche of anterior Hv (data not shown). Taken together our results possibly suggest that mGluR5 signaling may have a role in cell proliferation, survival and neuronal fate in a population of BrdU+ cells in adult telencephalic neurogenic zones.

## 4. Discussion

In the current study, we demonstrated that MK-801-treated zebrafish displaying impaired social interaction and increased anxiety levels, two common co-existing conditions in several neuropsychiatric illnesses (Allsop et al., 2014), were characterized by defective neurogenesis in the molecular level. In support, antimitotic drugs have been correlated with disrupted social recognition and memory (for refs García-Gómez et al., 2022). The above results are of particular interest since previous studies have also reported the presence of deficits in social communication and increased anxiety levels along with neurogenesis defects in various rodent models of neurological and neuropsychiatric diseases (Miller and Hen, 2015; Hong et al., 2019; Gioia et al., 2022), thus further validating this zebrafish pharmacological model as a useful tool for investigating the mechanistic link between dysregulated neurogenesis and comorbid social deficit and anxiety-like behavior. Interestingly, the decrease in the density of mitotically active cells in telencephalic and diencephalic proliferation zones was transient and followed by a significant increment of newborn cells in MK-801 treated zebrafish, 14 days after BrdU administration compared to their matching controls. In support, there is some evidence suggesting that reduced adult neurogenesis by stressful conditions might lead to increased responsiveness to future stressful events (Snyder et al., 2011). Indeed, following the cessation of MK-801 treatment, the restoration of social and anxiety phenotype was accompanied by increased densities of BrdU-labeled cells, the latter possibly representing a neuroplasticity mechanism counteracting the short-term effects. Such mechanism may act to upregulate the initially reduced population of radial glia and neuroblast cycling cells in MK-801-treated animals exhibiting social deficits and anxiety comorbidity. Future studies may determine the underlying causal mechanisms that link behavioral phenotype and adult neurogenesis.

At short-term survival, MK-801-treated zebrafish exhibited impaired sociability, with no preference for the social chamber during the social preference test, as also described in a previous study (Perdikaris and Dermon, 2023). In addition, MK-801-treated zebrafish displayed increased anxiety-like responses as indicated by their increased thigmotactic behavior in the OFT and their increased freezing duration and decreased exploration in the NTT, two well validated assays for estimation of fear and anxiety-like responses in zebrafish (Egan et al., 2009; Maximino et al., 2010). Interestingly, the above behavioral phenotype of MK-801-treated zebrafish was accompanied by disrupted dynamics of mitotic activity within neurogenic niches of key-areas of the SDM network, showing decreased densities of BrdU+ cells in the proliferation zones of dorsal and ventral telencephalon. It is possible that the different social behaviors, perhaps through changes in sensory input, influenced adult NSPCs activity in neurogenic niches of social processing areas, such as, amygdala, hippocampus, showing alterations in neurogenesis in response to a changing social environment. More specifically, regarding the dorsal telencephalon, the densities of mitotically active cells were decreased in the proliferation zones of Dm and Dld, two putative homolog structures of basolateral amygdala (BLA) and hippocampus of mammals (O'Connell and Hofmann, 2011), regulating both social behavior (Bickart et al., 2014; FeldmanHall et al., 2021) as well as fear and anxiety-like responses (Lal et al., 2018; Ghasemi et al., 2022). Our results further support previous evidence on decreased hippocampal NSPCs proliferation by NMDAR hypofunction (Maeda et al., 2007; Song et al., 2016; Ding et al., 2018). Moreover, in rodent animal models, chronic stress or deficits in social communication are characterized by the dysregulation of different stages in postnatal hippocampal neurogenesis including neural progenitor proliferation and differentiation (Malberg and Duman, 2003; Heine et al., 2004; Hong et al., 2019; Gioia et al., 2022).

The same pattern of downregulation of cell proliferation, following a 7-day MK-801 treatment, was observed in neurogenic zones of Vd and PPa, putative homolog structures of mammalian striatum and preoptic area (O'Connell and Hofmann, 2011). In contrast, this was not the case for the proliferation zone of Vv, a putative homolog structure of the lateral septum (Wullimann and Mueller, 2004) nor for the proliferation zone of Hv, the letter showing increased density of cycling cells following MK-801 treatment. Indeed, cell proliferation was specifically up-regulated in the ventricular zone of the anterior part of Hv, following MK-801 administration, indicating a region-specific mechanism that regulate cell proliferation in ventral hypothalamus. In support, different neuroplasticity genes are differentially expressed in zebrafish social phenotypes, in a niche-specific manner within the different nodes across the SDMN (Teles et al., 2016). Moreover, estradiol treatment had differential effects on cell proliferation pattern in adult zebrafish telencephalic ventricular zones (Makantasi and Dermon, 2014). Region-specific effects of MK-801 treatment may be related to the different populations of fast or slow dividing neuroblasts within adult dorsal and ventral neurogenic zones. In fact, the vast majority of dorsal telencephalic newborn cells represented slow-dividing radial glia cells (BrdU+/BLBP+) while ventral telencephalic and diencephalic areas were characterized by significantly smaller percentage of BrdU+/BLBP+ cells, in agreement to previous evidence supporting that these neurogenic niches are characterized by the presence of fast cycling (BrdU+/BLBP-) neuroblasts (Chapouton et al., 2010; März et al., 2010; Ampatzis et al., 2012). In addition, other studies in zebrafish have uncovered a key role of Wnt and Hedgehog signaling in regulating adult hypothalamic neurogenesis (Wang et al., 2012; Male et al., 2020) whereas Notch activity is known to control the transition between quiescence and neurogenesis along the telencephalic ventricular zone (Chapouton et al., 2010).

Glutamate and GABA regulate the quiescent to active state transition of NSPCs and subsequent activation of neurogenesis, by serving as “go/no go” signal (Trinchero and Schinder, 2020). Specifically, it has been suggested that neuronal hyperactivity, induced by the glutamate receptor agonist kainic acid, accelerated the depletion of the NSC pool in dentate gyrus by promoting its conversion in reactive astrocytes and impairing adult neurogenesis (Sierra et al., 2015). However, recent studies have shown that the action of glutamate in NSC proliferation is complex, since it may both promote and suppress NSPCs proliferation and survival depending on the type of glutamate receptor activated and glutamate concentration, with low concentration suggested to increase proliferation rate whereas high concentration is considered toxic (Luk et al., 2003; Giorgi-Gerevini et al., 2005; Erichsen et al., 2015; Zhang et al., 2021). In the present study, it is suggested that MK-801 suppressed the mitotic activity in telencephalic and diencephalic neurogenic zones of the SDM network, either acting directly on the distinct cell types within the proliferation zones or indirectly, affecting the regional neuronal excitability via metabotropic glutamate receptors. The latter hypothesis on a crucial role of mGluR5 is supported by their expression on proliferating cells within adult zebrafish telencephalic and diencephalic proliferation zones. To our knowledge, this is the first study in zebrafish, demonstrating mGluR5 expression on active proliferating progenitor cells, further supporting that dysregulated mGluR5 signaling may have a role in regulating cell proliferation. Indeed, MK-801-treated zebrafish exhibited strong mGluR5 immunoreactivity in brain parenchyma as well as elevated telencephalic and die-, mesencephalic mGluR5 protein levels (Perdikaris and Dermon, 2022), that possibly mediate the reduced mitotic activity, 15 h post-BrdU. However, 2 weeks following the cessation of MK-801 treatment, a significant upregulation of newborn cell densities was evident within telencephalic and diencephalic cell proliferation zones. Whether a possible restoration of mGluR5 signaling within the telencephalic and diencephalic proliferation zones is involved in the observed significant newborn cell increases, directly or indirectly (via regulation of circuit activity), needs to be further investigated.

Importantly, the presence of excessive IL-1β expression within the SDMN of MK-801-treated zebrafish (Perdikaris and Dermon, 2023) may represent an additional regulatory mechanism on cell proliferation. Indeed, our results showed that a percentage of proliferative progenitors express the pro-inflammatory cytokine IL-1β or are in close proximity with IL-1β^+^ cells, within the aforementioned telencephalic and diencephalic neurogenic niches, in consent to previous *in vitro* results reporting the expression of pro-inflammatory cytokines in human and adult rat neural progenitor cell lines (Klassen et al., 2003). Several studies have investigated the detrimental effects of inflammation on NSC proliferation (Ekdahl et al., 2003; Monje et al., 2003). More specifically, IL-1β reduced the proliferation of embryonic NPCs *in vitro* (Wang et al., 2007) and exerted the anti-proliferative effects of both acute and chronic stress *in vivo*, by activating the IL-1RI receptor, localized on adult hippocampal NPCs, and arresting cell cycle (Koo and Duman, 2008). Although, moderate concentrations of IL-1β can also exert beneficial effects in the survival of primary neurons *in vitro* (Brenneman et al., 1992) our data suggest that the neuroinflammatory response observed in MK-801-treated zebrafish, may be partly responsible for the decreases in cell proliferation in zebrafish telencephalic neurogenic zones. Moreover, it would be of importance to determine whether the normalization of IL-1β expression in telencephalic and diencephalic proliferation zones may contribute to the increased pattern of BrdU+ cells, 2 weeks following cessation of MK-801 treatment.

At long-term experiments, the number of BrdU+ cells remaining in the proliferation zones depends on the quiescent or active state, the proliferation rate of the mitotically active cell populations as well as on the migration of the postmitotic cells away from their birth site. As expected, in most areas studied, the density of BrdU+ cells was increased in both control and MK-81 long term survival groups, at 14 days post-BrdU, possibly due to further divisions of the short term labeled cells. Indeed, it is known that BrdU labeling can detect postmitotic cells up to three divisions (Hayes and Nowakowski, 2002). Interestingly, following the cessation of MK-801 treatment, the increases in the density of BrdU+ cells, was significantly higher than that of their matching control groups, indicating a compensatory rebound effect. Importantly, this increase was accompanied by the normalization and/or reversal of the social-deficit and anxiety-like behavior, further supporting the possible correlation between the regulation of neurogenesis and the manifestation of social dysfunction and increased anxiety levels. The increased density of BrdU+ cells observed in MK-801-LT group, could be either attributed to the increased survival of postmitotic cells or the increased proliferation rate of the mitotically active cells, following the cessation of NMDA receptor hypofunction. The latter hypothesis is further supported by the presence of BrdU+ clusters of 3 to 4 cells, within the telencephalic and diencephalic cell proliferation zones, but double labeling experiments with the proliferation marker PCNA will add important knowledge to support this assumption. Whether social behavior or reduced anxiety levels might contribute to shifting quiescent into a cycling state, or increase proliferation rate, thus increasing newborn cell density needs to be determined. These findings are toward the same direction with studies demonstrating that antidepressant treatment reversed the downregulation of cell proliferation as well as impaired sociability and behavioral despair, in animal models of ASD and stress (Czéh et al., 2001; Malberg and Duman, 2003; Gioia et al., 2022) and that adult newborn neurons’ activity has an effect on stress resilience (Anacker et al., 2018; Tunc-Ozcan et al., 2019).

Indeed, postmitotic newborn cells, exhibiting their neuronal commitment 14 days post-BrdU could potentially contribute to the restoration of excitatory and inhibitory neurotransmission, thus contributing to amelioration of social deficit and anxiety-like phenotype. Evidence for a role for adult newborn neurons contributing to the control of social behavior is provided from pairing neurogenic markers with markers of neural activity, showing that incorporation of newborn neurons is sensitive to the neuronal activity of the circuits, in response to specific experiences (Gheusi et al., 2009). Specifically, in zebrafish, a recent study showed that 15 min exposure to external social stimuli significantly increased of the percentage of active (expressing p56) adult born BrdU+ cells in dorsal telencephalic and preoptic regions of the zebrafish SDMN, 30 days post-BdrU (Dunlap et al., 2021). supporting the hypothesis that adult-born cells contribute to the regulation of social behavior. In agreement, it has been shown that synapses are already formed within 28 days of cell birth in adult zebrafish telencephalon (Rothenaigner et al., 2011). In this line, the present study provides evidence that adult neurogenesis contributes to a recovery from previous social deficit conditions, and that subpopulations of NSPCs (radial glial cells, neuroblasts) differentially influence the different nodes of SDMN activity. Further studies are needed to better characterize the possible brain regional specific mechanisms that link social deficits and anxiety-like behavior with dysregulated neurogenesis in MK-801-treated zebrafish. While the exact mechanisms remain to be identified, altered glutamatergic signaling and excessive pro-inflammatory responses are suggested to be involved and may represent targets for restoring neurogenesis and ameliorate social deficits and anxiety-like behavior.

## Data availability statement

The raw data supporting the conclusions of this article will be made available by the authors, without undue reservation.

## Ethics statement

The animal study was approved by Ethics committee of Patras University. The study was conducted in accordance with the local legislation and institutional requirements.

## Author contributions

CD and PPe conceived and designed experiments. PPe and PPr performed the experiments and analyzed the data. CD and PPe wrote and reviewed the manuscript. All authors contributed to the article and approved the submitted version.
