# Supplementary material for: Social withdrawal and anxiety-like behavior have an impact on zebrafish adult neurogenesis
Source: Front Behav Neurosci. 2023 Oct 16;17:1244075. doi: 10.3389/fnbeh.2023.1244075 (PMC10614005; doi:10.3389/fnbeh.2023.1244075)
Supplement: Supplementary file 1 [file Presentation_1.PPTX]

## Slide 1
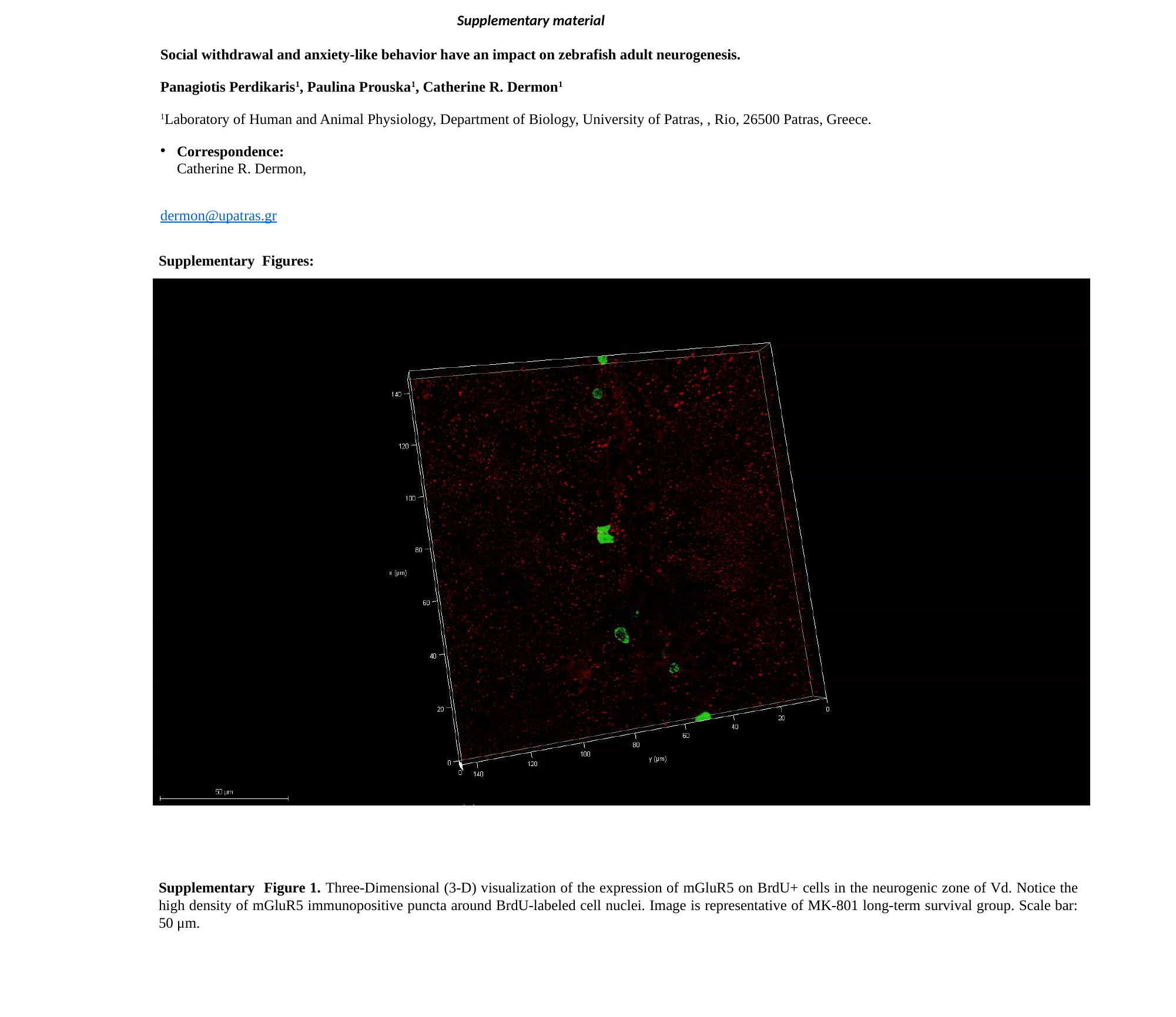

Supplementary material
Social withdrawal and anxiety-like behavior have an impact on zebrafish adult neurogenesis.
Panagiotis Perdikaris1, Paulina Prouska1, Catherine R. Dermon1
1Laboratory of Human and Animal Physiology, Department of Biology, University of Patras, , Rio, 26500 Patras, Greece.
Correspondence:
Catherine R. Dermοn,
dermon@upatras.gr
Supplementary Figures:
Supplementary Figure 1. Three-Dimensional (3-D) visualization of the expression of mGluR5 on BrdU+ cells in the neurogenic zone of Vd. Notice the high density of mGluR5 immunopositive puncta around BrdU-labeled cell nuclei. Image is representative of MK-801 long-term survival group. Scale bar: 50 μm.

## Slide 2
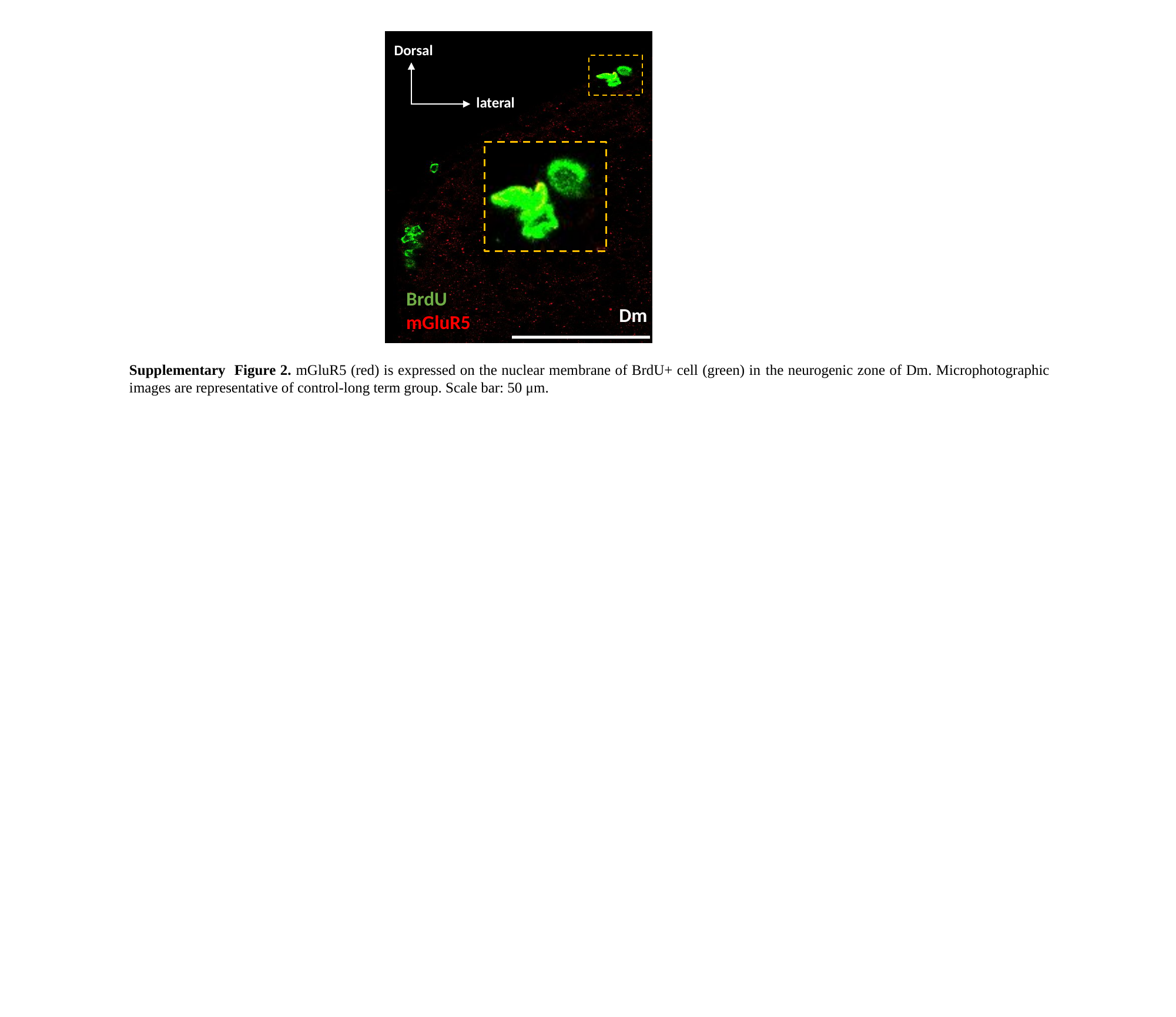

Dorsal
lateral
BrdU
mGluR5
Dm
Supplementary Figure 2. mGluR5 (red) is expressed on the nuclear membrane of BrdU+ cell (green) in the neurogenic zone of Dm. Microphotographic images are representative of control-long term group. Scale bar: 50 μm.
